# Association between intra-abdominal injured organs and abdominal compartment syndrome in patients with severe blunt trauma: A propensity score matched study using nationwide trauma registry in Japan

**DOI:** 10.1371/journal.pone.0286124

**Published:** 2023-05-23

**Authors:** Akira Komori, Hiroki Iriyama, Takako Kainoh, Makoto Aoki, Toshikazu Abe

**Affiliations:** 1 Department of Emergency and Critical Care Medicine, Tsukuba Memorial Hospital, Tsukuba, Japan; 2 Department of General Medicine, Juntendo University Faculty of Medicine, Tokyo, Japan; 3 Department of Health Services Research, Faculty of Medicine, University of Tsukuba, Tsukuba, Japan; 4 Advanced Medical Emergency Department and Critical Care Center, Japan Red Cross Maebashi Hospital, Maebashi, Japan; Bach Mai Hospital, VIET NAM

## Abstract

**Introduction:**

Abdominal compartment syndrome (ACS) after blunt abdominal trauma is a rare complication that requires early recognition and subsequent surgical intervention for optimal outcome. We aimed to investigate how differences in injured abdominal organs affect ACS development in patients with severe blunt abdominal trauma.

**Methods:**

This nested case-control study used a nationwide registry of trauma patients, namely, the Japan Trauma Data Bank (JTDB), and only included patients aged ≥ 18 years with blunt severe abdominal trauma, defined as an AIS score of abdomen ≥ 3, sustained between 2004 and 2017. Patients without ACS were used as control subjects and identified using propensity score (PS) matching. Characteristics and outcomes between patients with and without ACS were compared and logistic regression was used to identify specific risk factors for ACS.

**Results:**

Among 294,274 patients in the JTDB, 11,220 were eligible for inclusion before PS matching, and 150 (1.3%) developed ACS after trauma. PS matching led to the inclusion of 131 and 655 patients with and without ACS, respectively. Compared to controls, patients with ACS had higher number of injured organs in the abdomen and displayed a greater frequency of vascular and pancreatic injuries, need for blood transfusion, and disseminated intravascular coagulopathy, a complication of ACS. In-hospital mortality was higher in patients with ACS than those without ACS (51.1% vs. 26.0%, p < 0.01). Logistic regression analysis revealed that a higher number of injured organs in the abdomen [odds ratio (OR) (95% confidence interval [CI]): 1.76 (1.23–2.53)] and pancreatic injury [OR (95% CI): 1.53 (1.03–2.27)] were independently associated with ACS.

**Conclusions:**

Greater number of injured organs in abdomen and pancreatic injury are independent risk factors for the development of ACS.

## Introduction

Abdominal compartment syndrome (ACS) occurs following intraabdominal hypertension and is a rare complication that is associated with poor outcomes. Intraabdominal hypertension results in a series of pathophysiologic changes that begin with impaired regional blood flow, and these changes are associated with the systemic inflammatory response and whole-body ischemia-reperfusion secondary to blood loss and resuscitative procedures [1]. As decompressive laparotomy is recommended for the management of ACS [2], early recognition and subsequent treatment are essential for achieving optimal outcomes.

Risk factors for ACS after trauma have been reported by several studies and include severe trauma [3, 4], hemorrhagic shock [5, 6], and positive fluid balance [7, 8]. Most cases of ACS appear to be related to abdominal trauma; however, ACS has also been documented in cases without abdominal trauma [7, 9, 10]. As the pathophysiology of ACS varies with and without abdominal trauma, and categorizing all abdominal trauma as a single phenomenon may be inappropriate, it is prudent to separately assess risk factors. Therefore, based on data from a nationwide trauma registry in Japan, we aimed to investigate how differences in injured abdominal organs affect ACS development in patients with severe blunt abdominal trauma.

## Methods

### Study design, setting, and data source

This nested case-control study used information available in the Japan Trauma Data Bank (JTDB) for the period between 2004 and 2017. The JTDB is a nationwide trauma registry established in 2003 by the Japanese Association for the Surgery of Trauma and the Japanese Association for Acute Medicine to improve and ensure quality of trauma care in Japan [11]. A total of 272 hospitals, including more than 75% of the certified tertiary emergency medical centers in Japan, contributed to the JTDB in March 2018. Data captured in the JTDB includes patient and hospital information, such as patient demographics, Abbreviated Injury Scale (AIS) scores, Injury Severity Score, in-hospital procedures, complications, and clinical outcomes. Data collection is a part of routine clinical patient management.

### Study participants

Inclusion criteria were age ≥ 18 years and blunt trauma with severe abdominal injury, defined as an AIS severity score of 3 or greater for the abdomen. Patients with an AIS score of 6 (i.e., nonsurvivable injury) or those who died in the emergency department were excluded.

### Definitions

Primary outcome was the development of ACS, which was diagnosed based on the report of the physician in-charge; however, the database has no data on diagnostic criteria used for this purpose. The definitions of other complications included in this study concurred with those of the JTDB [11]. All emergency procedures were performed during resuscitation or initial management at the emergency department.

We categorized AIS codes representing injuries in the abdomen according to major organs involved, using AIS 90 Update 98, and presented the data as such. Injured organs included the blood vessels, digestive tract (stomach, duodenum, small intestine, colon, and rectum), mesentery, kidney, liver, pancreas, and spleen, among others.

### Statistical analysis

We selected eligible patients without ACS as controls to identify risk factors for ACS. First, we used propensity score (PS) matching to ensure that the ACS and control groups were balanced with respect to baseline characteristics and severity of trauma. PS calculations used the following variables that were selected based on clinical relevance and previous research, namely, age, sex, vital signs in the emergency department (Glasgow Coma Scale, systolic blood pressure, heart rate, and respiratory rate), and Injury Severity Score [11, 12]. Additionally, the treatment year was used for PS calculation, considering that the treatment strategy for severe abdominal trauma improved during the study period. Nearest neighbor propensity matching was performed at a 1:5 ratio and was based on averaged PS with a caliper of 0.2. The standardized mean difference of variables was used to evaluate balance after PS matching, and a standardized mean difference of > 0.1 defined as meaningful imbalance.

Next, we compared injury regions, injured organs in the abdomen, comorbidities, emergency procedures or interventions, concomitant complications, and outcomes between the ACS and control groups. Continuous variables are presented as median and interquartile range and were compared using the Mann–Whitney *U* test because none of the variables were normally distributed. Categorical variables are presented as numbers and percentages and were compared using the Chi-square test.

After comparison of baseline characteristics, logistic regression was employed to identify risk factors for developing ACS. We assessed the multicollinearity of variables using the variance inflation factor with the tolerance value set at less than 5. We also carefully examined clinically plausible interactions; however, no meaningful interactions were found among the variables tested. We included type of injured organs in the abdomen with an AIS severity score ≥ 3 as candidate risk factors in model 1. In model 2, we included the number of injured organs in the abdomen with an AIS severity score ≥ 3 as candidate risk factor. In model 3, we used both type and number of injured organs, and added complications of coagulopathy, as disseminated intravascular coagulopathy (DIC) and thrombocytopenia, in models 4. All models were adjusted for variables such as transfusion within 24 h after arrival at the emergency department and chronic hepatic conditions, including liver cirrhosis and chronic hepatitis, as they are known to be associated with ACS [7, 13]. Transcatheter arterial embolization as an emergency procedure was also used for adjusting all models considering that transcatheter arterial embolization means the vascular injury of abdominal organs and potentially affects ACS development.

For sensitivity analysis, we also used the estimated propensity scores as weights and used an inverse probability weighting (IPW) model to generate a weighted cohort. A logistic regression was subsequently performed on the weighted cohort to identify risk factors for developing ACS using the same variables in the primary analysis.

All probability values were two-sided, and p < 0.05 was considered statistically significant. We performed statistical analyses using the Stata software, version 15.1 (Stata Corp., TX, USA).

### Ethical approval and consent to participate

The study protocol was reviewed and approved by the Research Ethics Committee of the Tsukuba Memorial Hospital (IRB No. R03-09-05). Given the retrospective and anonymized nature of this study, the Research Ethics Committee of the Tsukuba Memorial Hospital waived the need to obtain informed consent from the study participants. JTDB administrators also provided permission to use their database. All methods were performed in accordance with the relevant guidelines and regulations.

## Results

**Fig 1:**
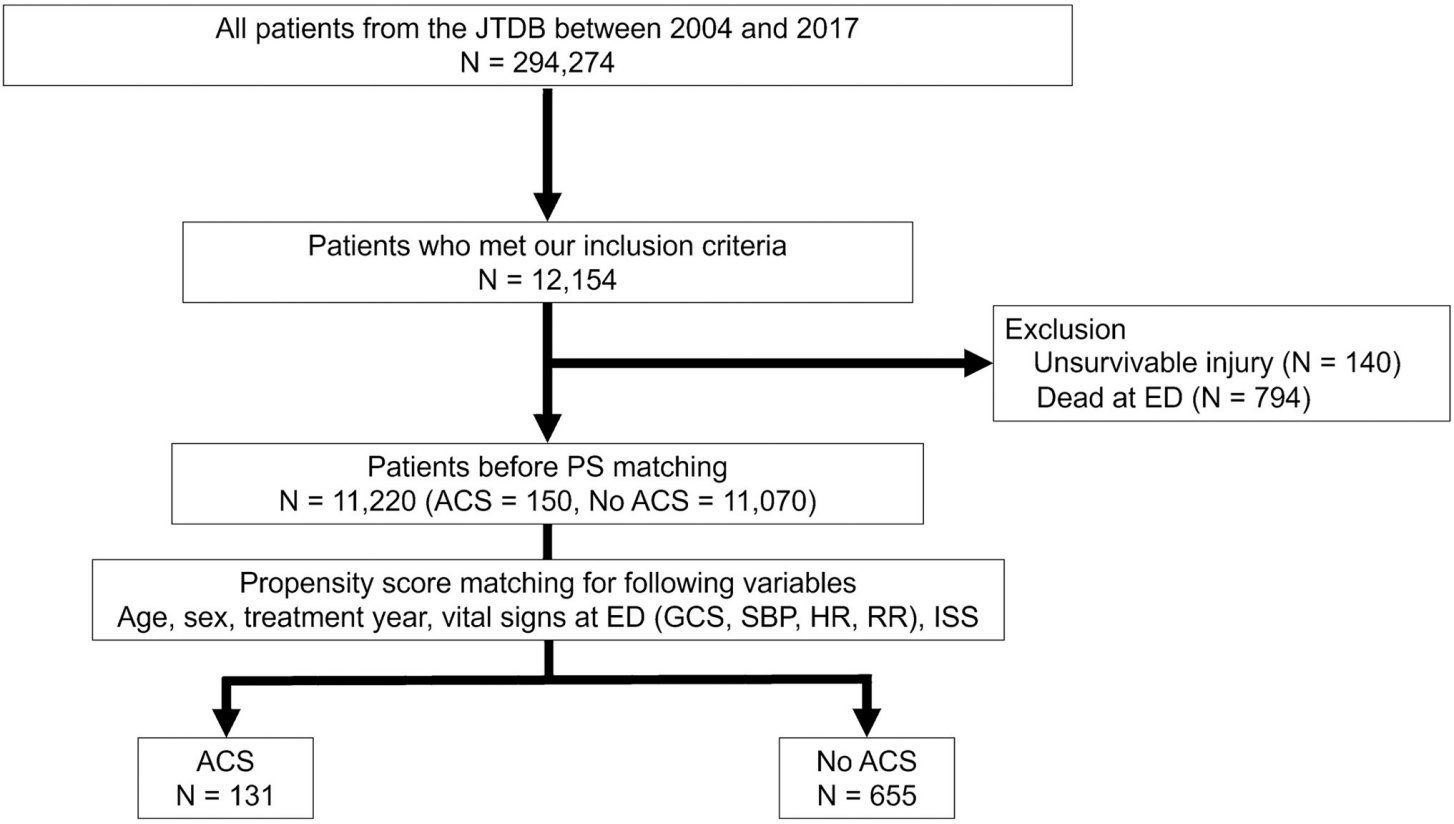
Patient selection for this study. ACS, abdominal compartment syndrome; ED, emergency department; GCS, Glasgow coma scale; HR, heart rate; ISS, Injury Severity Score; JTDB, Japan Trauma Data Bank; PS, propensity score; RR, respiratory rate; SBP, systolic blood pressure.

Among 294,274 trauma patients registered in the JTDB between 2004 and 2017, 11,220 patients were eligible for inclusion in this study (Fig 1). Of these, 150 (1.3%) patients developed ACS after trauma, and after PS matching, data from 131 patients with ACS was compared with that from 655 patients without ACS. Median age of the study participants was 55 years (interquartile range, 36–70), 624 (79.4%) were male, and median Injury Severity Score was 32 (interquartile range, 20–43). PS matching did not reveal any meaningful imbalance of variables between patients with and without ACS (Table 1).

**Table 1:** Basic characteristics and interventions in severe abdominal trauma patients with and without ACS.

		ACS	No ACS	SMD	*P*—value
		N = 131	N = 655		
Age		54 (34–70)	58 (38–70)	0.051	0.59
Gender (male)		105 (80.2)	519 (79.2)	0.023	0.81
Treatment year	2004–2009	29 (22.1)	136 (20.8)	0.018	0.93
	2009–2013	68 (51.9)	350 (53.4)		
	2014–2017	34 (26.0)	169 (25.8)		
Vital signs at emergency department	GCS	13 (9–15)	14 (8–15)	0.023	0.34
	SBP	92 (74–123)	99 (73–121)	0.013	0.84
	HR	103 (84–127)	103 (85–125)	0.012	0.90
	RR	25 (20–30)	24 (20–30)	0.013	0.85
Injury Severity Score		27 (17–45)	32 (21–42)	0.001	0.74
Injured body region (AIS ≥ 3)	Head	28 (21.4)	198 (30.2)		0.04
	Thorax	59 (45.0)	393 (60.0)		<0.01
	Spine	8 (6.1)	61 (9.3)		0.24
	Upper extremity	7 (5.3)	46 (7.2)		0.48
	Lower extremity including the pelvis	45 (34.4)	213 (32.5)		0.68
Injured organ in abdomen (AIS ≥ 3)	Blood vessel	38 (29.0)	98 (15.0)		<0.01
	Digestive duct	21 (16.0)	83 (12.7)		0.30
	Mesentery	15 (11.5)	90 (13.7)		0.48
	Kidney	23 (17.6)	79 (12.1)		0.08
	Liver	43 (32.8)	165 (25.2)		0.07
	Pancreas	12 (9.2)	8 (1.2)		<0.01
	Spleen	22 (16.8)	132 (20.2)		0.38
	Others	13 (9.9)	110 (16.8)		0.05
Number of injured organs in the abdomen (AIS ≥ 3)	1	59 (45.0)	428 (65.3)		<0.01
	2	35 (26.7)	162 (24.7)		
	≥3	37 (28.2)	65 (9.9)		
Comorbidities	Ischemic heart diseases	2 (1.5)	16 (2.4)		0.52
	Heart failure	1 (0.8)	2 (0.3)		0.44
	Hypertension	18 (13.7)	96 (14.7)		0.79
	Asthma	1 (0.8)	10 (1.5)		0.50
	COPD	2 (1.5)	5 (0.8)		0.40
	Liver cirrhosis	7 (5.3)	10 (1.5)		<0.01
	Chronic hepatitis	4 (3.1)	13 (2.0)		0.44
	Peptic ulcer	1 (0.8)	11 (1.7)		0.44
	Inflammatory bowel diseases	1 (0.8)	5 (0.8)		0.99
	DM	12 (9.2)	46 (7.0)		0.39
	Obesity	1 (0.8)	3 (0.5)		0.65
	Stroke	1 (0.8)	15 (2.3)		0.26
	Dementia	1 (0.8)	7 (1.1)		0.75
	Malignancies	2 (1.5)	10 (1.5)		0.99
	Anticoagulant use	1 (0.8)	0		0.03
	Hemodialysis	3 (2.3)	6 (0.9)		0.17
Emergency procedures	Oral intubation	92 (70.2)	319 (48.7)		<0.01
	Ventilator use	69 (52.7)	208 (31.8)		<0.01
	Aortic cross-clamping	3 (2.3)	20 (3.1)		0.64
	REBOA	23 (17.6)	50 (7.6)		<0.01
	Thoracentesis	2 (1.5)	7 (1.1)		0.65
	Chest drainage	27 (20.6)	152 (23.2)		0.52
	Blood transfusion within 24 h	96 (73.3)	340 (51.9)		<0.01
	Transcatheter arterial embolization	66 (50.4)	179 (27.3)		<0.01
	Vasopressor use	45 (34.4)	111 (17.0)		<0.01
	Skeletal traction	4 (3.1)	35 (5.3)		0.27
	External skeletal fixation	11 (8.4)	42 (6.4)		0.41
	Other emergency bone fixation	4 (3.1)	22 (3.4)		0.86
Primary surgeries	Craniotomy	2 (1.5)	8 (1.2)		0.78
	Thoracotomy	6 (4.6)	43 (6.6)		0.39
	Celiotomy	81 (61.8)	266 (40.6)		<0.01
	Bone reduction and fixation	8 (6.1)	80 (12.2)		0.04

Propensity matched by age, gender, year, vital signs at emergency department (GCS, SBP, HR, RR), and Injury Severity Score.

Continuous variables were compared using the Mann–Whitney U test. Categorical variables were compared using the Chi-square test.

ACS, abdominal compartment syndrome; SMD, standardized mean difference; GCS, Glasgow coma scale; SBP, systolic blood pressure; HR, heart rate; RR, respiratory rate; AIS, Abbreviated Injury Scale score; COPD, chronic obstructive pulmonary disease; DM, diabetes mellitus; REBOA, resuscitative endovascular balloon occlusion of the aorta

Vascular and pancreatic injuries were more common in patients with ACS than in those without ACS [29.0% vs. 15.0%; p < 0.01 for vascular injury, and 9.2% vs. 1.2%; p < 0.01 for pancreatic injury, respectively]. Compared to those without ACS, number of injured organs in the abdomen was higher and liver cirrhosis was more frequent in patients with ACS (5.3% vs. 1.5%, p < 0.01). Only one patient with ACS was prescribed an anticoagulant; none in the without ACS group used this medication. There were no significant differences in other comorbidities between patients with and without ACS.

Next, patients with ACS were more frequently provided blood transfusion than those without ACS (73.3% vs. 51.9%, p < 0.01). Celiotomy was the most commonly performed primary surgery in both groups, and it was more frequently indicated in patients with ACS than in those without ACS (61.8% vs. 40.6%, p < 0.01).

Concomitant complications that were more common in ACS patients than in those without ACS are listed in the S1 Table. DIC and coagulation disorders (38.9% vs. 7.5%, p < 0.01) and thrombocytopenia (26.7% vs. 3.7%, p < 0.01) were more frequent in patients with ACS than in those without ACS.

In-hospital mortality was higher in patients with ACS than in those without ACS (51.1% vs. 26.0%, p < 0.01, Table 2), and while all patients with ACS were admitted to intensive care units (ICU), only 94.7% patients without ACS were admitted to the ICU. There were no significant differences in length of hospital stay or ICU stay between patients with and without ACS.

**Table 2:** Outcomes of severe abdominal trauma patients with and without ACS.

		ACS	No ACS	*P*—value
		N = 131	N = 655	
Disposition at discharge	Died (in-hospital mortality)	67 (51.1)	170 (26.0)	<0.01
	Transfer	33 (25.4)	287 (44.0)	
	Home	28 (21.5)	194 (29.8)	
	Others	2 (1.5)	1 (0.2)	
ICU admission		131(100.0)	620 (94.7)	0.06
Length of hospital stay		24 (3–60)	26 (7–55)	0.62
ICU stay		12 (2–47)	13 (2–35)	0.94

Continuous variables were compared using the Mann–Whitney U test. Categorical variables were compared using the Chi-square test.

Missing: disposition at discharge: 4, length of hospital stay: 10, ICU stay: 96.

ACS, abdominal compartment syndrome; ICU, intensive care unit.

**Fig 2:**
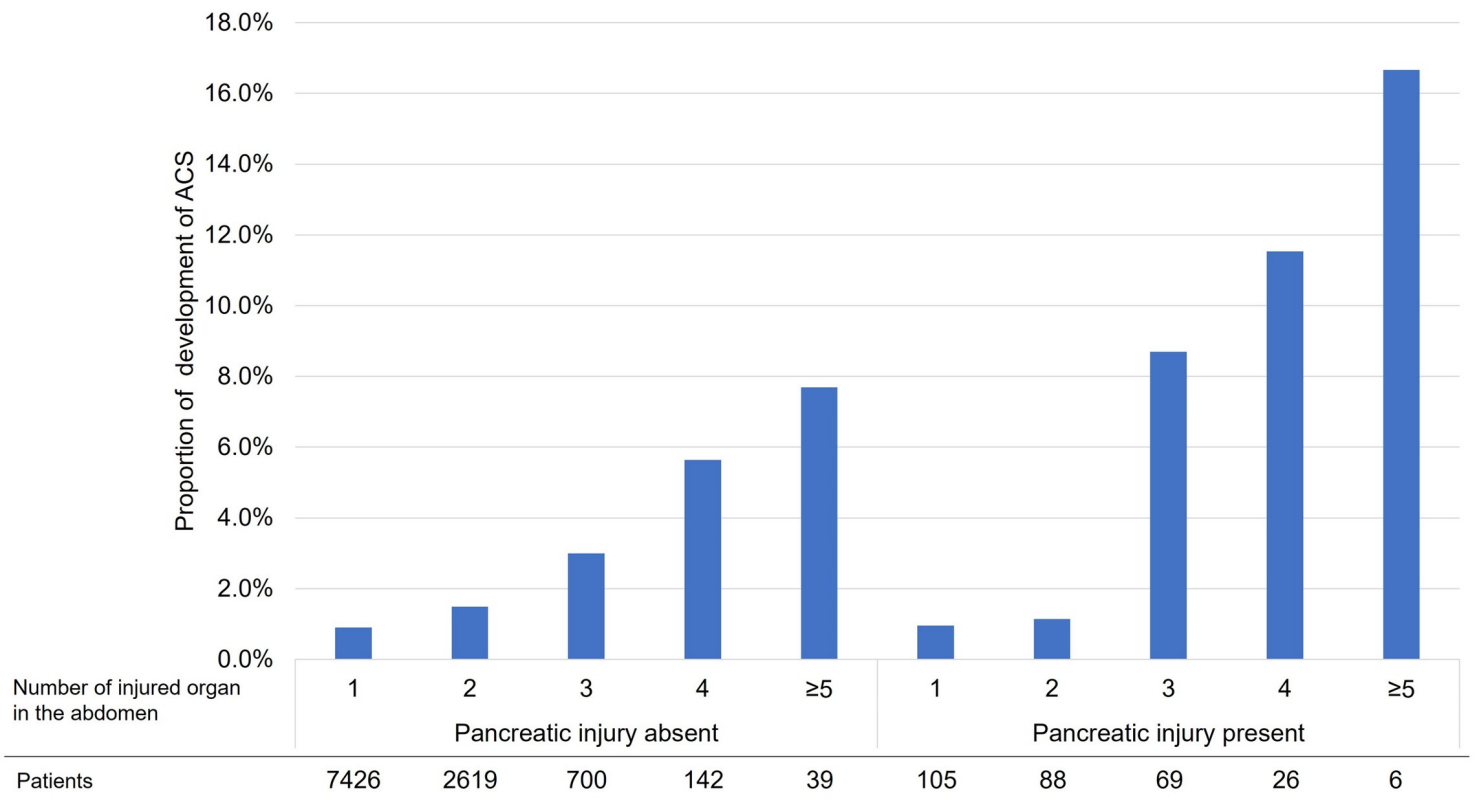
Proportion of development of ACS by number of injured organs in the abdomen and presence or absence of pancreatic injury (N = 11,220, before PS matching). ACS, abdominal compartment syndrome; PS, propensity score.

Table 3 shows the results of logistic regression models to identify risk factors for developing ACS. Number of injured organs in the abdomen was consistently associated with ACS in models 2, 3, and 4. Finally, only injury to the pancreas was consistently associated with ACS, while vascular, kidney, and liver injury were not associated with ACS models 3 and 4, although an association was seen in model 1. Injury to the mesentery, spleen, and digestive tract were not associated with ACS. DIC and coagulation disorders [odds ratio (95% confidence interval): 4.11 (2.16–7.82), p < 0.01] and thrombocytopenia [3.00 (1.40–6.39), p < 0.01] were associated with ACS. The results remained stable in IPW analyses (S2 Table). The relationship between the presence of pancreatic injury and the number of abdominal organ injuries to the incidence of ACS is shown in Fig 2.

**Table 3:** Logistic regression models to identify risk factors associated with ACS.

	Model	OR (95% CI)			
		1	2	3	4
Injured organ in the abdomen (AIS ≥ 3)	Blood vessel	1.42 (1.17–1.73)		1.20 (0.97–1.50)	1.22 (0.96–1.54)
	Kidney	1.32 (1.08–1.61)		1.11 (0.89–1.39)	1.19 (0.94–1.51)
	Liver	1.28 (1.08–1.53)		1.11 (0.91–1.35)	1.15 (0.93–1.42)
	Mesentery	1.10 (0.87–1.38)		0.90 (0.70–1.17)	0.91 (0.70–1.19)
	Pancreas	1.97 (1.39–2.78)		1.67 (1.17–2.39)	1.53 (1.03–2.27)
	Spleen	1.11 (0.91–1.35)		0.96 (0.77–1.19)	0.96 (0.77–1.21)
	Digestive tract	1.31 (1.06–1.63)		1.14 (0.90–1.43)	1.14 (0.89–1.46)
	Others	1.00 (0.80–1.27)		0.87 (0.67–1.11)	0.91 (0.70–1.19)
Number of injured organs in the abdomen (AIS ≥ 3)			2.01 (1.56–2.58)	1.72 (1.22–2.43)	1.76 (1.23–2.53)
DIC and coagulopathy					4.11 (2.16–7.82)
Thrombocytopenia					3.00 (1.40–6.39)

All models are adjusted by transfusion < 24 h on arrival at emergency department, hepatic diseases (liver cirrhosis and chronic hepatitis), and performed transcatheter arterial embolization.

OR, odds ratio; CI, confidential interval; AIS, Abbreviated Injury Scale score; DIC, disseminated intravascular coagulopathy

## Discussion

Abdominal trauma is a major cause of ACS, and using data from a large database, we show that the prevalence of ACS among patients with severe blunt abdominal trauma was low (at 1.3%) in the Japanese national trauma registry. Further, interestingly, ACS development was associated with the number of organs injured in the abdomen rather than the type of organ injured, and injury to most organs in the abdomen, except for the pancreas, was not associated with ACS. Coagulopathy, including DIC, was also associated with ACS, and it is possible that these complications represent a cause-and-effect phenomenon. Thus, focusing on the number of abdominal organs injured may help clinicians stratify patients based on risk of ACS development during the early stages of trauma care.

Our analysis of a national trauma registry identified an independent association between the number of injured organs in the abdomen and development of ACS. This result is as expected because hemorrhage is the main pathology in trauma, and multiple organ injuries, as well as severity of injury, are related to greater hemorrhage [14]. In addition, hemorrhaging ascites directly increase intraabdominal pressure. Thus, copious fluid resuscitation to counter massive hemorrhage induces capillary leak and intestinal edema [15], both of which are risk factors for ACS secondary to increased intraabdominal pressure [12]. Therefore, clinicians should be aware of the risk of developing ACS when patients present with multiple organ injuries in the abdomen.

Anatomical features of the injured organ may be important for the development of ACS, and in our cohort, only pancreatic injury was associated with the development of ACS. The pancreas is located in the retroperitoneum and is typically present near the great vessels, i.e., the inferior vena cava, portal vein, and abdominal aorta, which not only make surgical repair of the pancreatic injury challenging [16, 17] but also, thereby, delay hemorrhage control. Moreover, physiological and biochemical features of pancreatic trauma, such as leakage of pancreatic enzymes, can contribute to the development of both ACS and pancreatitis.

In our study, coagulopathy, including DIC, was associated with ACS, but a cause-and-effect relationship, i.e., whether DIC associated with trauma precedes ACS, could not be established because the JTDB does not capture data about the timing complication onset. It is rational to expect that, pathophysiologically, DIC may have preceded ACS, with subsequent trauma not only exacerbating hemorrhage but also contributing to the increased intraabdominal pressure and resulting in the development of ACS. Nevertheless, it must be noted that the reverse could also be true. Very few previous studies have discussed the association between DIC and development of ACS; specifically, a single case series showed that two patients developed ACS following DIC [18]. Although trauma-induced coagulopathy might be present in the early phase after trauma [19], it is unknown whether coagulopathy in the early phase progresses to DIC [20, 21].

Preventing complications will undoubtedly improve mortality [11], and we confirm that ACS is one of the most severe complications of severe abdominal trauma because more than half of the patients who developed ACS died. Although primary injury of the abdominal organs or DIC induced by trauma may be unmodifiable, clinicians should pay attention when patients present with these risk factors as it can help to potentially retard or stop the development of ACS, consider avoid excessive positive fluid balance [5, 6, 22].

### Limitations

Our study has some limitations. First, unmeasured potential confounders could have affected the results, and although the amount of fluid or blood transfused are risk factors for developing ACS [6, 22], our database did not contain this data. Nonetheless, we adjusted the logistic regression models using a related variable, i.e., blood transfusion within 24 h of admission. Second, celiotomy may be a potential risk factor because a few previous studies have reported abdominal surgery to be a risk factor for ACS [5, 8]. However, we did not include celiotomy in the logistic regression analysis because time elapsed between celiotomy and ACS was unknown. In addition, ACS rapidly develops after injury and can present early, often within 3–6 h of admission to the emergency department [1, 6, 7, 23]; hence, presumably, ACS could have developed before primary surgery in some cases. Third, potential information bias, due to the ACS diagnosis being based on the reports of the physician in-charge, along with possible underdiagnosis, cannot be ruled out. However, the prevalence of ACS associated with trauma in previous reports ranges from 0 to 6.2% [5, 8, 9, 24], which is consistent with our results. In addition, as most of the institutions participating in the JTDB were nationally certified emergency centers, we believe that most of the patients received appropriate trauma care. Fourth, some injured organs in the abdomen may have been underreported because the AIS was not designed to assess the combined effects of multiple organ injuries at the same site. This limitation may have affected the results.

## Conclusion

We investigated the role of abdominal organ injury in the development of ACS in patients with severe blunt abdominal trauma and show that greater number of injured organs is an independent risk factor for ACS. Additionally, injury to the pancreas was independently associated with the development of ACS.

## Supporting information

S1 Table Complications of severe abdominal trauma patients with and without ACS. Categorical variables were compared using the Chi-square test. ACS: abdominal compartment syndrome, ARDS: acute respiratory distress syndrome, GI: gastrointestinal, DIC: disseminated intravascular coagulopathy, MOF: multiple organ failure.
(DOCX)

S2 Table Logistic regression models using inverse probability weighting to identify risk factors associated with ACS. All models are adjusted by transfusion < 24 h on arrival at emergency department, hepatic diseases (liver cirrhosis and chronic hepatitis) and performed transcatheter arterial embolization. OR, odds ratio; CI, confidential interval; AIS, Abbreviated Injury Scale score; DIC, disseminated intravascular coagulopathy.
(DOCX)
