# Supplementary material for: Association between intra-abdominal injured organs and abdominal compartment syndrome in patients with severe blunt trauma: A propensity score matched study using nationwide trauma registry in Japan
Source: PLoS One. 2023 May 23;18(5):e0286124. doi: 10.1371/journal.pone.0286124 (PMC10204983; doi:10.1371/journal.pone.0286124)
Supplement: S1 Table — Categorical variables were compared using the Chi-square test. ACS: abdominal compartment syndrome, ARDS: acute respiratory distress syndrome, GI: gastrointestinal, DIC: disseminated intravascular coagulopathy, MOF: multiple organ failure. (DOCX) [file pone.0286124.s001.docx]

|  |  | ACS | No ACS |  |
| --- | --- | --- | --- | --- |
|  |  | N =131 | N = 655 | *P* - value |
| Central nerve system | Diabetes insipidus | 1 (0.8) | 4 (0.6) | 0.84 |
|  | higher brain dysfunction | 4 (3.1) | 24 (3.6) | 0.73 |
| Circulation | Lethal arrhythmia | 1 (0.8) | 2 (0.3) | 0.44 |
|  | Refractory shock | 26 (19.9) | 15 (3.0) | <0.01 |
|  | Acute renal failure | 24 (18.3) | 14 (2.1) | <0.01 |
| Respiratory | Lung edema | 7 (5.3) | 7 (1.1) | <0.01 |
|  | Atelectasis | 20 (15.3) | 24 (3.7) | <0.01 |
|  | Pneumonia | 23 (17.6) | 39 (6.0) | <0.01 |
|  | Pulmonary embolism | 3 (2.3) | 5 (0.8) | 0.11 |
|  | ARDS and respiratory failure | 22 (16.8) | 18 (2.8) | <0.01 |
| Gastroenterology and hepato-biliary | Ulcer and Upper GI bleeding | 3 (2.3) | 7 (1.1) | 0.26 |
|  | Ileus | 7 (5.3) | 9 (1.4) | <0.01 |
|  | Pancreatitis | 1 (0.8) | 0 | 0.03 |
|  | Cholecystitis | 1 (0.8) | 5 (0.8) | 0.99 |
|  | Hyperbilirubinemia and Liver failure | 18 (13.7) | 8 (1.2) | <0.01 |
| Bone and joint | Compartment syndrome | 1 (0.8) | 7 (1.1) | 0.75 |
| Hematologic | DIC and coagulopathy | 51 (38.9) | 49 (7.5) | <0.01 |
|  | Thrombopenia (< 50000) | 35 (26.7) | 24 (3.7) | <0.01 |
| Others | Bacteremia | 11 (8.4) | 10 (1.5) | <0.01 |
|  | Sepsis or MOF | 29 (22.1) | 13 (2.0) | <0.01 |
|  | Abdominal abscess | 9 (6.9) | 10 (1.5) | <0.01 |
|  | Urinary tract infection | 11 (8.4) | 14 (2.1) | <0.01 |
|  | Wound infection | 21 (16.0) | 28 (4.3) | <0.01 |
|  | Hypothermia (< 35℃) | 16 (12.2) | 10 (1.5) | <0.01 |
