# Supplementary material for: Association between intra-abdominal injured organs and abdominal compartment syndrome in patients with severe blunt trauma: A propensity score matched study using nationwide trauma registry in Japan
Source: PLoS One. 2023 May 23;18(5):e0286124. doi: 10.1371/journal.pone.0286124 (PMC10204983; doi:10.1371/journal.pone.0286124)
Supplement: S2 Table — All models are adjusted by transfusion < 24 h on arrival at emergency department, hepatic diseases (liver cirrhosis and chronic hepatitis) and performed transcatheter arterial embolization. OR, odds ratio; CI, confidential interval; AIS, Abbreviated Injury Scale score; DIC, disseminated intravascular coagulopathy. (DOCX) [file pone.0286124.s002.docx]

|  |  | OR (95% CI) | | | |
| --- | --- | --- | --- | --- | --- |
|  | Model | 1’ | 2’ | 3’ | 4’ |
| Injured organ in the abdomen (AIS ≥ 3) | Blood vessel | 1.39 (1.14–1.69) |  | 1.22 (0.98–1.52) | 1.23 (0.97–1.57) |
|  | Kidney | 1.30 (1.04–1.63) |  | 1.17 (0.90–1.51) | 1.26 (0.95–1.67) |
|  | Liver | 1.37 (1.13–1.67) |  | 1.25 (0.99–1.58) | 1.21 (0.94–1.56) |
|  | Mesentery | 1.18 (0.90–1.54) |  | 1.06 (0.81–1.39) | 1.12 (0.86–1.47) |
|  | Pancreas | 2.05 (1.39–3.02) |  | 1.86 (1.21–2.87) | 1.84 (1.12–3.04) |
|  | Spleen | 1.02 (0.82–1.26) |  | 0.93 (0.73–1.17) | 0.92 (0.72–1.18) |
|  | Digestive tract | 1.03 (0.82–1.30) |  | 0.93 (0.73–1.18) | 0.89 (0.70–1.14) |
|  | Others | 0.99 (0.78–1.26) |  | 0.91 (0.70–1.18) | 0.96 (0.73–1.27) |
| Number of injured organs in the abdomen (AIS ≥ 3) |  |  | 1.83 (1.36–2.46) | 1.49 (1.02–2.18) | 1.51 (1.03–2.21) |
| DIC and coagulopathy |  |  |  |  | 4.16 (2.20–8.12) |
| Thrombocytopenia |  |  |  |  | 2.73 (1.27–5.85) |
